# Insights from the 2023 May measurement month campaign in Newfoundland and Labrador, Canada: A cross-sectional study

**DOI:** 10.1097/MD.0000000000042522

**Published:** 2025-05-16

**Authors:** Stephen D. Coombs, Ross T. Tsuyuki, Stephanie W. Young, Neil Poulter, Tiffany A. Lee

**Affiliations:** a Memorial University School of Pharmacy, St. John’s, Canada; b University of Alberta Faculty of Medicine and Dentistry, Edmonton, Canada; c Imperial College London National Heart and Lung and School of Public Health, London, UK.

**Keywords:** hypertension, May measurement month, mass screening, Newfoundland and Labrador, Canada

## Abstract

May measurement month (MMM) is a global blood pressure (BP) screening campaign that aims to emphasize the importance of BP measurement and identify those who require intervention/follow-up for elevated BP. The objective of this regional analysis in Newfoundland and Labrador (NL), Canada, was to examine the proportion of individuals screened with elevated BP, including those with and without a history of hypertension (HTN). This cross-sectional study was completed in accordance with the global MMM protocol. All consenting adults ≥18 years old were eligible to take part. Data collection took place in 28 community pharmacies across the province of NL. Descriptive statistics were analyzed and associations between elevated BP and covariates of interest were determined using logistic regression. A total of 384 participants took part in this study, with a mean age of 54.4 years (standard deviation 18.2); 66.1% (n = 254) of participants were female and 41.4% (n = 159) had known HTN. A complete set of 3 BP readings were recorded for a total of 375 participants and therefore, these participants were included in the analysis. Elevated BP was observed in 21.9% (n = 82) of participants, including 13.5% of those who had no history of HTN (i.e., 30 of 222). Known HTN and diabetes were statistically significant predictors of elevated BP in the multivariate regression model. Regional implementation of the MMM campaign in NL helped to identify a relatively large proportion of individuals with elevated BP, including those with no history of HTN. Targeted measures are needed to achieve BP targets among individuals with hypertension and diabetes in the province.

## 1. Introduction

The Canadian province of Newfoundland and Labrador (NL) has the highest rates of hypertension (HTN) in the country, with nearly 30% of adults reported to have high blood pressure.^[1]^ NL also has high rates of mortality due to heart disease and stroke, and has earned a D-grade on health system performance.^[2,3]^ A recent C.D. Howe report provides further insight into reasons for poor health system performance, with NL ranking last on access to care and second to last in preventative care^[4]^ both of which are necessary strategies to prevent and manage HTN and its sequelae. Of importance, HTN is well-documented as the major risk factor for cardiovascular (CV) morbidity and mortality, accounting for approximately half of all CV and stroke related events.^[5–7]^ Despite its well-documented risks, HTN is largely asymptomatic, earning the label the “silent killer.”^[1]^ Therefore, early detection and management are pivotal to reducing the burden of hypertension-related complications, including heart disease, stroke, and kidney failure.^[5,8–10]^

An aging population, high rates of obesity, and lifestyle-related risks are factors (e.g., high salt intake, sedentary behaviors) that contribute to the burden of hypertension in NL, making it a vital area of focus for public health interventions.^[3,11]^ A cross-sectional study conducted by members of our team in 2022 identified that nearly 27% of individuals screened had elevated blood pressure (BP) and nearly 20% of participants who reported no history of HTN had elevated BP.^[12]^ Consequently, continued surveillance is necessary to monitor and assess the burden of HTN in the province. While large-scale surveys like the Canadian Health Measures Survey (CHMS) provide valuable insights into HTN trends, they have notable limitations in capturing current epidemiological patterns related to HTN awareness, detection, treatment, and control. The CHMS, conducted periodically, may not fully reflect recent changes in public health initiatives, healthcare access, or treatment advancements. Additionally, its reliance on a national sampling framework can overlook regional variations, such as the disproportionately high rates of HTN in NL. In addition, the 2022 to 2024 CHMS did not include data collection sites from in NL in its administration.^[13]^ These limitations highlight the need for localized screening efforts to provide a more comprehensive understanding of HTN nuances and to tailor interventions effectively.

Global initiatives such as the May measurement month (MMM) campaign have emphasized the importance of HTN screening. MMM, an annual event led by an international group, May Measure Month (https://maymeasure.org/about/), has successfully mobilized healthcare professionals and volunteers worldwide to screen millions of individuals. MMM has raised awareness about the prevalence of high BP and encouraged communities to prioritize CV health.^[14,15]^ Furthermore, the campaign underscores the impact of coordinated, large-scale screening efforts, particularly in low-resource settings, to ensure ongoing surveillance and appropriate distribution of healthcare resources. In NL, we worked with community pharmacists from across the province to implement the MMM 2023 campaign. Leveraging the accessibility and trust associated with pharmacists enabled us to make BP screening more convenient and widespread, particularly for individuals who may not regularly interact with healthcare providers via traditional means.

Screening for HTN in community and primary health care settings plays an important role in addressing the risks associated with high BP.^[5,7,10,16]^ These environments offer accessible, cost-effective, and scalable platforms for identifying individuals at risk and support the initiation of timely interventions. Opportunistic BP screening ensures that high-risk populations, particularly those in underserved or resource-limited settings, are not overlooked.^[5]^ In fact, community-based screening for elevated BP has been identified as a key strategy to address the global burden of disease, and to reduce the risk of CV and stroke related complications associated with elevated BP.^[17–19]^ Furthermore, a recent survey of Canadian health experts conducted by the Heart & Stroke Foundation of Canada identified routine BP screening by community practitioners, including pharmacists and paramedics, as a key strategy “to support individuals with HTN or who are at risk of developing high BP.”^[10]^

This paper reports on our local adoption of the 2023 MMM campaign in NL and presents updated information pertaining to the burden of elevated BP as well as lifestyle-related risk factors prevalent in the province. The primary objective of this regional analysis was to examine the proportion of individuals screened with elevated BP, including those with and without a history of HTN. This work aims to inform policy and practice, and to improve local screening efforts.

## 2. Methods

### 2.1. Research design and setting

This cross-sectional study was completed using a modified version of the global MMM protocol. Twenty-eight community pharmacies engaged in research data collection during the 2023 MMM campaign across the island portion of NL, including 12 rural sites and 16 urban sites. Pharmacies were considered ideal data collection sites; they are accessible to foot traffic, have a private consultation room and space to engage in BP measurement and consultation, and support data collection and BP measurement by highly qualified healthcare professionals.

### 2.2. Implementation of the MMM blood pressure screening campaign

With support from the local research team, each community pharmacy designated a site champion (i.e., pharmacist or senior pharmacy student) to oversee the implementation of the campaign and research project. Pharmacists and pharmacy students at each site completed a structured training program, including the virtual Pan American Health Organization course on accurate automated BP measurement (https://campus.paho.org/en/course/Blood-Pressure-Measurement), and participated in a study launch meeting with the research team. The principal investigator (TL) also conducted virtual site visits with each pharmacy student to ensure adherence to BP measurement best practices, appropriate set-up of consultation rooms and BP measuring devices, as well as to provide support and troubleshoot, as needed.

A member of the pharmacy team (pharmacist or pharmacy student) asked each participant a series of questions to collect information about covariates of interest, including demographic and clinical characteristics, lifestyle information, medical conditions, and relevant medications, prior to BP measurement. The pharmacist or pharmacy student then proceeded to collect 3 BP and pulse readings from each participant using a validated, Hypertension Canada recommended, automated office blood pressure device provided by the research team.

Three BP measurements were performed to increase the reliability of the readings. BP was measured following the procedures recommended by Hypertension Canada and the MMM organization: no smoking or caffeine 30 minutes before BP measurement; the participant must be seated (with their feet flat on the floor and their back supported) in a quiet consultation room for 5 minutes before initiation of measurements; an appropriately sized BP cuff applied to the participant’s bare arm, supported by a table at the level of the heart; no talking during the measurement; the provider must leave the room during measurement; and 3 readings taken 1 minute apart, with the last 2 readings averaged and communicated to the patient.^[20]^

Elevated BP was defined as an average systolic BP (SBP) ≥ 140 mm Hg and/or an average diastolic BP (DBP) ≥ 90 mm Hg. In accordance with Hypertension Canada recommendations, for individuals with diabetes, elevated BP was defined as an average systolic BP (SBP) ≥ 130 mm Hg and/or an average DBP ≥ 80 mm Hg.^[20]^ If a patient was identified as having elevated BP, they were referred to their primary care provider (family physician or nurse practitioner), or to a walk-in clinic or emergency room if they did not have a primary care provider. Pharmacists could also manage elevated BP based on the provincial Standards of Practice: Prescribing by Pharmacists.^[21]^ Individuals with evidence of hypertensive urgency (SBP ≥ 180 mm Hg and/or DBP ≥ 110 mm Hg) or hypertensive emergency (urgency plus symptoms) were referred to the nearest emergency room.^[20]^ Following BP measurement, the pharmacist or pharmacy student engaged each participant in conversation about strategies to lower blood pressure (e.g., healthy diet, increased physical activity) and provided patient friendly reading materials, as needed.

### 2.3. Study population and sample size

All consenting adults ≥18 years of age were eligible to take part; there were no exclusion criteria. Each participant had to visit one of the research sites on just 1 occasion for BP screening to be included; an individual did not have to be “attached” to one of the community pharmacy research sites (i.e., a regular patient of the pharmacy) to take part. It should be noted that intervention and follow-up were not part of this study. However, pharmacists were encouraged to practice at the top of their scope and engage in follow-up as deemed appropriate based on the participant’s BP readings.

A sample size of 323 was calculated using the formula for frequency in a population with a provincial adult population size of 440,000, an anticipated frequency of elevated BP equal to 30%, and a 5% margin of error at a 95% confidence level. Our goal was to recruit 350 participants.

### 2.4. Recruitment

A variety of marketing materials (print, web, and social media) were used to promote the study. For example, a recruitment poster was developed and shared on various social media platforms (e.g., Facebook, Instagram, and X) and through paid Facebook advertising. The poster included a link to a website that housed information about the MMM research project in NL, including a list of pharmacy sites and BP screening times. Pharmacy personnel were also permitted to recruit patients from their site to take part; however, we discouraged the recruitment of individuals with known HTN, as we did not want this group to be overrepresented in the study sample.

### 2.5. Data collection

Data collection began on May 17, 2023, to coincide with World Hypertension Day and continued until May 31, 2023. Using a modified version of the MMM questionnaire, information pertaining to participant socio-demographic characteristics, medical history, clinical characteristics (e.g., weight, height, date of last BP measurement), lifestyle factors and the use of antihypertensives and other selected medications was collected. Reported heights and weights were used to calculate body mass index in kg/m^2^. Pharmacy personnel also measured and recorded 3 seated BP and pulse readings for each participant using a validated automated office BP device (WatchBP Office^®^, Microlife AG, Widnau, Switzerland) and correct size cuff provided by the study team. All data were collected anonymously and entered into the secure online data platform (REDCap) maintained by EPICORE at the University of Alberta.

### 2.6. Data analysis

Analyses were performed using the Statistical Package for the Social Sciences, version 29.0. Descriptive statistics were used to describe the study population, including mean and standard deviation for continuous variables and frequency counts and percentages for categorical variables. Measures of association used data from only those participants with a complete set of 3 BP readings. Logistic regression techniques were used to investigate the relationship between our binary dependent variable, elevated BP, and various participant characteristics using univariate analyses. Statistically significant variables in the univariate analyses were entered into the multivariate model and adjusted for sex. Odds ratios and their associated 95% confidence intervals, as well as the final model goodness of fit (C-statistic), are reported. Chi square tests were also performed for categorical variables and 2-sided independent *t* tests for continuous variables (age and body mass index) when comparing participants with and without elevated BP. Statistical significance was set at *P* ≤ .05.

### 2.7. Data management

Data management was facilitated by the EPICORE Centre, University of Alberta. The data manager built the data collection tool in REDCap and provided each community pharmacy site with access to the data entry fields using their unique ID code; however, only selected research team members (TL and SC) had access to the provincial dataset to perform data analysis. Data for this study were shared with the MMM lead researchers for Canada (RT) and the global MMM Organization (NP). As such, data collected for this NL study are included in national and international analyses. The results of the Canadian dataset are published in the Canadian Pharmacists Journal^[22]^ and manuscript preparation for the global MMM dataset is ongoing.

### 2.8. Ethical considerations

This study was reviewed and approved by the Newfoundland and Labrador Health Research Ethics Board (HREB Reference #2023.069). Informed consent was obtained from participants by the pharmacist and/or pharmacy student at each research site. All participant data collected for MMM was anonymous and not traceable to the individual participants.

## 3. Results

A total of 384 participants took part in this study. An average of 13 participants (range 3–35) were screened at each of the 28 pharmacy sites. Most sites enrolled participants on 1 day only (i.e., May 17—World Hypertension Day); however, some sites offered screening on several days (range 1–6 days). The mean age of participants was 54.4 years (standard deviation 18.2), 66.1% (n = 254) were female, and 41.4% (n = 159) had known hypertension. Further details regarding participant characteristics are reported in Table 1.

**Table 1:** Participant characteristics (n = 384).

	Frequency, n (%)
Age in years, mean (SD)	54.4 (18.2)
Age categories, yr	
18–34	74 (19.3%)
35–44	42 (10.9%)
45–54	54 (14.1%)
55–64	83 (21.6%)
65 and older	131 (34.1%)
Female sex	254 (66.1)
Mean BMI, kg/m^2^ (SD)*	29.3 (6.08)
Current smoker	54 (14.1)
Alcohol use (at least once weekly)	102 (26.6)
Physical inactivity	139 (36.2)
BP measured within the last 12 months†	316 (82.3)
Known hypertension	159 (41.4)
Comorbidities	
History of MI	23 (6.0)
History of stroke	9 (2.3)
Heart failure	13 (3.4)
Diabetes	60 (15.6)
CKD	8 (2.1)
Selected medications	
Antihypertensive(s)	159 (41.4)
Statin	119 (31.0)
ASA	59 (15.4)
Oral anticoagulant	25 (6.5)

ASA = acetylsalicylic acid, BMI = body mass index, CKD = chronic kidney disease, MI = myocardial infarction, SD = standard deviation.

* Missing data for 11 participants.

† Missing data for 2 participants.

A total of 375 participants had a complete set of 3 BP readings and were included in the analysis of BP. The majority of these participants (n = 309, 82.4%) reported having their BP measured within the last 12 months. However, 5 individuals with known HTN and 3 individuals with diabetes indicated that their last BP measurement was performed more than 12 months prior. Elevated BP was observed in 82 (21.9%) participants, including 30 (13.5% of 222) of those who indicated they did not have a history of HTN and 52 (34.0% of 153) participants with known HTN. Elevated BP was observed in other subgroups of interest (see Table 2). Two participants met the criteria for hypertensive urgency and were identified as having a SBP ≥ 180 mm Hg and/or DBP ≥ 110 mm Hg and were referred for urgent care.

**Table 2:** Subgroups of interest with elevated blood pressure.

	Proportion (%)
Sex	
Female	51/246 (20.7)
Male	31/129 (24.0)
Current smoker	14/52 (26.9)
Current alcohol user	32/190 (16.8)
Known hypertension	52/153 (34.0)
Taking 1 or more antihypertensives	50/153 (32.7)
Taking oral anticoagulant	5/24 (20.8)
Diabetes	27/59 (45.8)
History of MI	7/22 (31.8%)
History of stroke	4/9 (44.4)
Heart failure	5/13 (38.5)
CKD	3/8 (37.5)

CKD = chronic kidney disease, MI = myocardial infarction.

Statistically significant associations between the presence of elevated BP and independent variables (e.g., age, sex, and comorbidities) are presented in Table 3. In the univariate analyses, increasing age, physical inactivity, known hypertension, and diabetes were associated with elevated BP. Known hypertension and diabetes remained statistically significant in the multivariate model with a C-statistic of 0.771. Further details regarding differences between those with elevated BP and those without are presented in Table 4, where statistically significant findings are consistent with the univariate analyses.

**Table 3:** Relationship between participant variables and elevated BP.

	Univariate			Multivariate		
	OR	95% CI	*P* value	OR	95% CI	*P* value
Age	1.029	1.013–1.044	<.001	1.011	0.993–1.029	.236
Female sex	0.827	0.497–1.374	.463	0.832	0.484–1.431	.506
Current smoker	1.382	0.708–2.696	.343			
Alcohol use	0.651	0.360–1.175	.154			
Physical inactivity	1.661	1.009–2.735	.046	1.334	0.782–2.275	.290
BMI	1.035	0.994–1.078	.098			
Known hypertension	3.295	1.979–5.486	<.001	2.223	1.228–4.024	.008
Diabetes	4.004	2.222–7.216	<.001	2.666	1.423–4.993	.002
Previous MI	1.730	0.681–4.396	.250			
Previous stroke	2.954	0.775–11.263	.113			
Heart failure	2.313	0.736–7.272	.151			
CKD	2.187	0.512–9.351	.291			

CI = confidence interval, CKD = chronic kidney disease, BMI = body mass index, MI = myocardial infarction, OR = odds ratio.

**Table 4:** Comparison of participants with elevated BP (n = 82) to those without (n = 293).

	Elevated BP	Normal BP	*P* value
	Frequency, n (%)	Frequency, n (%)	
Mean age, yr (SD)	61.1 (14.5)	52.5 (18.8)	<.001
Female sex	51 (62.1)	195 (66.6)	.463
Mean BMI, kg/m^2^ (SD)	30.2 (5.7)	28.9 (5.9)	.097
Physical inactivity	37 (45.1)	97 (33.1)	.045
Current smoker	14 (17.1)	38 (13.0)	.342
Alcohol use (at least once weekly)	17 (20.7)	84 (28.7)	.152
Known hypertension	52 (63.4)	101 (34.5)	<.001
Diabetes	27 (32.9)	32 (10.9)	<.001
Prior MI	7 (8.5)	15 (5.1)	.244
Prior stroke	4 (4.9)	5 (1.7)	.097
Heart failure	5 (6.1)	8 (2.7)	.141
CKD	3 (3.7)	5 (1.7)	.280

BMI = body mass index, CKD = chronic kidney disease, MI = myocardial infarction.

## 4. Discussion

In the province of NL, we were able to successfully implement the MMM campaign and recruit 384 participants over a period of 2 weeks to take part in the BP screening initiative with more than one-third of our participants aged 65 years and older. The results of our study indicate a number of key findings. Nearly 22% of participants screened had elevated BP, which includes those with and without a history of HTN. Additionally, 13.5% of participants who indicated they had never been diagnosed with HTN had elevated BP. These findings highlight the need for a targeted HTN case finding service in the province. Such a service could be modeled after the National Health Service Community Pharmacy Blood Pressure Check Service which aims to support risk identification and prevention of CV disease.^[23]^

Our study also revealed that a large number of individuals with known HTN are not at target, using the definition of an average SBP ≥ 140 mm Hg and/or an average DBP ≥ 90 mm Hg as our reference. In the current study, 34% of participants with known HTN were not at target, which was very similar to 36% in 2022.^[12]^ The 2025 Hypertension Canada guidelines, which are set to be published later this year, as well as the 2024 European Society of Cardiology Guidelines for the management of elevated BP and HTN suggest a BP target of ≤130/80 mm Hg for most individuals.^[24]^ Consequently, a larger proportion of our participants would have met the definition of elevated BP if we had used this as our reference point. Establishing BP control is critical to avoiding HTN-related complications, including heart and kidney disease.^[5,8–10]^ Adjustment of antihypertensives and addressing medication nonadherence are 2 strategies that can help to establish this control, both of which should become a priority for community pharmacist practice. Of note, our study did not evaluate guideline concordant prescribing of antihypertensives and therefore, we are unable to report on whether this contributed to our findings.

Female participants, who represented more than two-thirds of our sample, were quite similar to males in terms of the proportion with elevated BP (approximately 21% and 24%, respectively). Damianaki and colleagues in their study of MMM in Switzerland pharmacies make a similar observation, suggesting that females are more likely to be screened in pharmacies than their male counterparts.^[25]^ This is an important finding in and of itself, since HTN awareness, detection, treatment, and control among females is a priority for Hypertension Canada.^[26]^

The large proportion of individuals with elevated BP who have diabetes in our study is also quite concerning. In fact, nearly 50% of participants with diabetes had BP readings above the Hypertension Canada recommended target of ≤130/80 mm Hg. This finding is consistent with the results of our 2022 study,^[12]^ suggesting that gains have not been made in this area. Furthermore, in our multivariate analysis, we determined that the odds of having elevated BP was 2.6 times more likely in participants with diabetes. Given that CV disease is the leading cause of death in this population, adequately treating HTN to attain recommended BP targets is crucial.^[27]^ Screening programs in community pharmacies can successfully help to identify individuals with diabetes who have not achieved their BP target and sets the stage for future pharmacist-led BP screening initiatives in this population.

This study had a number of important strengths. Our ability to engage 28 community pharmacy sites in data collection was instrumental in helping us achieve our target sample size of 350 participants. To optimize recruitment and increase the accessibility of BP screening, we aligned this project with the global MMM campaign and offered BP screening at different sites throughout NL from May 17 to 31, 2023. Furthermore, all study personnel (pharmacists and pharmacy students) received the same training on BP measurement from the study team; sites were provided with an automated, office-grade BP measurement device recommended by Hypertension Canada and BP measurement took place in a private and quiet consultation room to ensure adherence to best practices for BP measurement.

This study also has some limitations. Convenience sampling and opportunistic BP screening limit the generalizability of study findings^[28]^ and may introduce some selection bias. Therefore, the proportion of participants with elevated BP should not be interpreted as the population prevalence of HTN. We also did not collect information on race and ethnicity, which are important considerations when examining the relationship between participant characteristics and elevated BP. Furthermore, a single set of BP measurements were collected for each participant, which is not sufficient for making a diagnosis of HTN or for identifying persistently elevated BP. We were unable to rule out the presence of “white-coat” or masked HTN. Despite this, elevated BP prompted pharmacists to refer patients for further assessment and management. Information pertaining to medical and medication history was self-reported by participants, which may have introduced reporting bias. Although we used Hypertension Canada definitions for HTN case finding (i.e., BP ≥ 140/90 mm Hg or ≥130/80 mm Hg for individuals with diabetes), we were unable to identify those individuals with high CV risk whose treatment target is SBP < 120 mm Hg^[20]^ due to our inability to collect information pertaining to lipid values from patients and perform CV risk assessment.

In terms of knowledge translation and mobilization, our team has been working with Hypertension Canada to develop a policy brief for the provincial government to improve the detection, treatment, and control of high BP in NL. To improve access to care and enable preventative care in NL, both of which were critical needs identified in a recent C.D. Howe report,^[4]^ action is needed to better utilize and support all highly qualified community-based providers to optimize BP screening and management services. Participation in the annual MMM campaign may also help to raise awareness about the importance of healthy lifestyle behaviors as well as BP measurement.

## 5. Conclusion

A large number of individuals with elevated BP were identified during the MMM 2023 campaign in NL, including those with and without an established diagnosis of hypertension. The results of our study also reinforce prior knowledge that individuals with diabetes are not meeting their BP targets. Given the unique demographic and lifestyle-related risk factors prevalent in the province, accessible screening programs delivered by community pharmacists may provide a feasible strategy for early detection, and subsequent management, of the “silent killer” we know as HTN.

## Acknowledgments

This work was supported by a research start-up grant provided to Dr Tiffany Lee by the Vice-President Research at Memorial University. The authors thank the pharmacists and pharmacy students who participated in the data collection for this study. We also wish to thank the EPICORE Centre, University of Alberta, for support with data management and Dr Omar Bdair (Biostatistician, Memorial University) for consultation regarding statistical analyses.

## Author contributions

**Conceptualization:** Stephen D. Coombs, Ross T. Tsuyuki, Stephanie W. Young, Neil Poulter, Tiffany A. Lee.

**Data curation:** Stephen D. Coombs.

**Formal analysis:** Stephen D. Coombs, Tiffany A. Lee.

**Methodology:** Stephen D. Coombs, Ross T. Tsuyuki, Stephanie W. Young, Tiffany A. Lee.

**Project administration:** Tiffany A. Lee.

**Resources:** Ross T. Tsuyuki, Tiffany A. Lee.

**Supervision:** Tiffany A. Lee.

**Writing – original draft:** Stephen D. Coombs, Tiffany A. Lee.

**Writing – review & editing:** Ross T. Tsuyuki, Stephanie W. Young, Neil Poulter, Tiffany A. Lee.
